# RNA decay in processing bodies is indispensable for adipogenesis

**DOI:** 10.1038/s41419-021-03537-7

**Published:** 2021-03-17

**Authors:** Ryotaro Maeda, Daisuke Kami, Akira Shikuma, Yosuke Suzuki, Toshihiko Taya, Satoaki Matoba, Satoshi Gojo

**Affiliations:** 1 Department of Cardiovascular Medicine, Graduate School of Medicine, Kyoto Prefectural University of Medicine, Kyoto, Japan; 2 Department of Regenerative Medicine, Graduate School of Medicine, Kyoto Prefectural University of Medicine, Kyoto, Japan

**Keywords:** Multivesicular bodies, RNA decay

## Abstract

The RNA decay pathway plays key regulatory roles in cell identities and differentiation processes. Although adipogenesis is transcriptionally and epigenetically regulated and has been thoroughly investigated, how RNA metabolism that contributes to the stability of phenotype-shaping transcriptomes participates in differentiation remains elusive. In this study, we investigated Ddx6, an essential component of processing bodies (PBs) that executes RNA decay and translational repression in the cytoplasm and participates in the cellular transition of reprogramming. Upon adipogenic induction, Ddx6 dynamically accumulated to form PBs with a binding partner, 4E-T, at the early phase prior to emergence of intracellular lipid droplets. In contrast, preadipocytes with Ddx6 knockout (KO) or 4E-T knockdown (KD) failed to generate PBs, resulting in significant suppression of adipogenesis. Transcription factors related to preadipocytes and negative regulators of adipogenesis that were not expressed under adipogenic stimulation were maintained in Ddx6-KO and 4E-T-KD preadipocytes under adipogenic induction. Elimination of Dlk1, a major negative regulator of adipogenesis, in 3T3L1 Ddx6-KO cells did not restore adipogenic differentiation capacity to any extent. Similar to murine cells, human primary mesenchymal stem cells, which can differentiate into adipocytes upon stimulation with adipogenic cocktails, required DDX6 to maturate into adipocytes. Therefore, RNA decay of the entire parental transcriptome, rather than removal of a strong negative regulator, could be indispensable for adipogenesis.

## Introduction

Cellular identity is driven by genomic, epigenomic, transcriptomic, and proteomic heterogeneity. In stem cells or progenitors, intrinsic entities that are responsible for environmental cues make fate decisions to either maintain stemness or differentiate into progenies^1^. As multiomics technology has developed, comprehensive regulatory networks related to cell trajectory have been elucidated in detail. The epigenome, transcriptional regulation, and protein degradation have been extensively investigated, but how RNA metabolism contributes to the mechanisms has not been clarified^2^. Transcript expression does not directly correlate with transcription rates^3^ but rather is determined by the balance between transcription rates and decay rates^4^. Although RNA stability differs considerably among various transcripts^5^, the decay rates of some transcripts are conserved among different species^6^, which has led to the discovery of an RNA decay regulon^7^. The molecular aspects of RNA decay are well defined, whereas the phenotypic linkages to cellular phenotypes and physiological functions are poorly characterized.

RNA decay-dependent removal of previous cellular transcripts may be a common feature of various cellular transitions, as we have previously reported that reprogramming to induce pluripotent stem cells requires eradication of the unique transcriptomes of parent cells^8^. In addition to RNA decay, control of mRNA levels is executed by translational repression. The molecular machinery that silences targeted mRNAs via RNA decay or translational repression is composed of the RNA-induced silencing complex (RISC)^9^, which consists of microRNAs that recognize their target mRNAs through partial complementarity with the help of Argonaute proteins^10^. During translation^11^, the RNA decay machinery can form closed loops in the target mRNA through a bridge that is established between the 3′-end deadenylation complex and the 5′ m7G-cap by binding to eIF4E, which is mediated by Ddx6 and 4E-T^12^. On the other hand, translational repression occurs at the initial step of translation via PABP displacement^13^; recruitment of a translational repressor, such as Ddx6^14^; and dissociation of eIF4A^15^. For RNA metabolism, cells contain membraneless organelles, which are flexible biological condensates composed of many proteins. Such organelles include various ribonucleoprotein (RNP) granules; processing bodies (PBs), which are maintained by essential factors including Ddx6, 4E-T, and LSM14A^16^; and stress granules in the cytoplasm. Along with RISCs, the spatial and temporal association or dissociation of these effector molecules, such as Ddx6 and 4E-T, enables fine-tuning of the transcriptome to ensure that it is appropriate for the environment and that it maintains homeostasis in the body.

Ddx6 belongs to the DEAD-box family of RNA helicases involved in RNA decay via activation of decapping and translational repression. During erythrocyte differentiation, degradation of mitochondria in enucleated mature reticulocytes is triggered by reticulocyte 15-lipoxygenase (r15-LOX), and r15-LOX mRNA is translationally repressed in PBs of erythroid precursor cells by Ddx6 in combination with hnRNP K and hnRNP E1, which recognize the differentiation control element in the 3′-untranslated region (3′-UTR)^17^. In macrophages, TNF-α is regulated by translational repression induced by recognition of AU-rich elements (AREs) residing in the 3′-UTR of TNF-α mRNA by tristetraprolin (TTP) and Ddx6^18^. Messenger RNPs in PBs are apparent standbys that respond to extracellular stimuli or appropriately timed events. Ddx6 binds to 4E-T, Pat1, and Edc3 in an exclusive manner, suggesting that the partner may determine the fate of the RNP, such as translational repression in the case of 4E-T, deadenylation for RNA decay in the case of Pat1, or decapping for RNA decay in the case of Edc3^19^.

Information on the molecular mechanism of adipogenesis has been acquired by culturing adipocyte precursor cell lines, such as the 3T3L1 (unipotent cells) and 10T1/2 (pluripotent fibroblasts) cell lines, in a defined adipogenic cocktail containing insulin, dexamethasone, and 3-isobutyl-1-methylxanthine that stimulates cAMP-dependent protein kinase^20^. Transcriptional regulation of adipogenesis is well characterized; at least two waves of transcription factors are involved in the process. The first wave of factors is dominated by CCAAT/enhancer-binding protein (C/EBP) β/δ, and the second is centered around peroxisome proliferator-activated receptor (PPAR) γ and C/EBPα^21^. A variety of adipogenic signals activate the transcription factors of the first waves, such as AP-1, KLF4, and KLF6, in addition to C/EBPβ and C/EBPδ, which in turn associate with transcription factor hotspots and coordinate enhanceosome formation and chromatin remodeling leading to differentiation into mature adipocytes. Positive regulators of the adipogenic program are antagonized by several types of negative regulators enriched in preadipocytes, such as GATA2, GATA3, KLF2, KLF7, and Dlk1 (also known as Pref1), which are fully repressed during adipogenesis^22^. In addition to *trans*-acting factors, *cis*-acting regulatory elements within mRNA, often within 3′-UTRs, might govern the fate of cytoplasmic mRNAs through RNA decay pathways^23^.

We have previously demonstrated that Ddx6-dependent RNA decay is indispensable for the reprogramming that induces pluripotent stem cells^8^. Extending the findings to other biological processes, we hypothesized that RNA decay may play an essential role in differentiation from precursors and tested this hypothesis through in vitro adipogenesis. The role of RNA metabolism in in vitro adipogenesis was investigated by silencing Ddx6 and 4E-T, which are essential constituents of PBs. This study elucidates a novel aspect of adipogenesis in the context of the RNA decay regulon.

## Results

### PBs and the expression of mesenchymal genes during adipogenesis

**Fig. 1:**
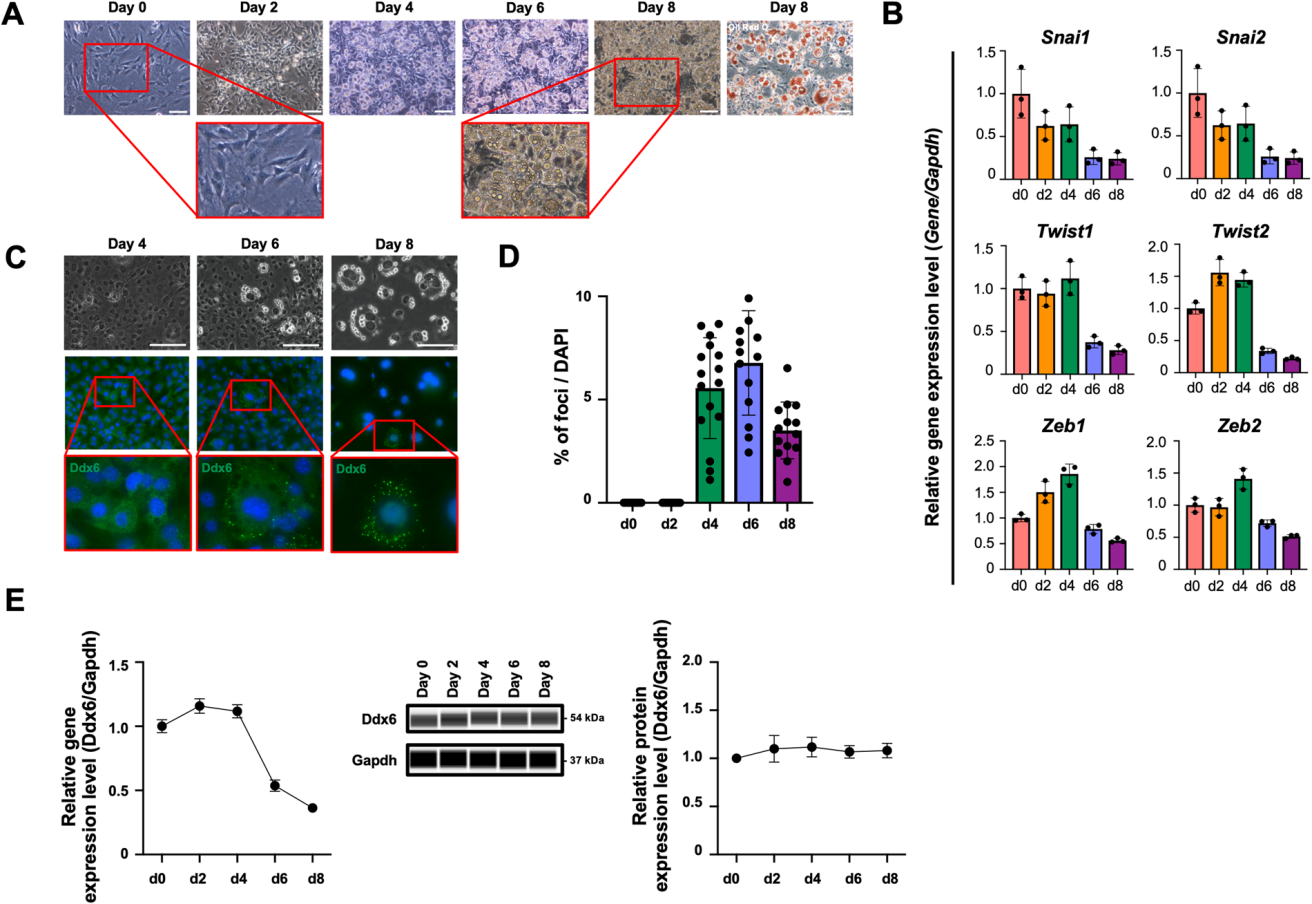
Adipogenic differentiation of 3T3L1 preadipocytes. **A** Phase-contrast microscopy images during adipogenesis. 3T3L1 preadipocytes were differentiated into adipocytes using an adipogenic differentiation cocktail containing dexamethasone, IBMX, and insulin. On day 8, the cells were fixed and stained with oil red O. The scale bar is 100 μm. **B** RT-qPCR analysis of gene expression in 3T3L1 preadipocytes during adipogenesis. Individual RNA expression levels were normalized to Gapdh expression levels. The error bars indicate the SDs (*n* = 3). **C** Immunocytochemistry images during adipogenesis. Formation of Ddx6 foci was observed. The scale bar is 50 μm. Nuclei were stained with DAPI. **D** Ratio of the number of cells with Ddx6 foci to the number of DAPI-positive cells each day following adipogenic induction analyzed in three sessions. Independent researchers chose 7–5 microscopic fields in a session at random. **E** Protein (*n* = 4) and gene (*n* = 6) expression analysis of Ddx6 during adipogenesis. The error bars indicate the SDs.

The preadipocyte cell line 3T3L1 was used in this study to investigate the role of RNA metabolism in adipogenesis induced by standard differentiation cocktails^24^. Preadipocytes reached confluence on day 2 after adipogenic induction, and adipocytes laden with lipid droplets appeared at approximately day 4 (Fig. 1A). The fatty acid droplets were stained with oil red O on day 8. The expression of mesenchymal genes was assayed because these genes shape the mesenchymal phenotype of 3T3L1 cells. The levels of Snai1 and Snai2 continuously and gradually decreased, whereas the levels of Twist 1/2 and Zeb 1/2 were increased on day 4 and abruptly decreased on day 6 (Fig. 1B). RNA decay occurs in PBs, which are specialized membraneless organelles for RNA metabolism that are involved in the onset of differentiation of stem cells^8^; PBs of 3T3L1 cells during adipogenesis were visualized by staining with Ddx6, an essential protein of PBs. Ddx6-positive foci appeared on day 4 of adipogenesis, and their numbers gradually decreased until day 8 (Fig. 1C, D). The expression of Ddx6 mRNA was decreased after 4 days of adipogenic induction; however, the protein levels of Ddx6 were maintained during adipogenesis (Fig. 1E), suggesting that free Ddx6, which does not participate in PB formation, may be less stable than PBs. The PBs formed at the same time that the expression of mesenchymal transcription factors decreased on day 4 of adipogenic induction, suggesting that the role of RNA metabolism during adipogenesis should be investigated.

### Inhibition of adipogenesis by Ddx6 knockout (KO)

**Fig. 2:**
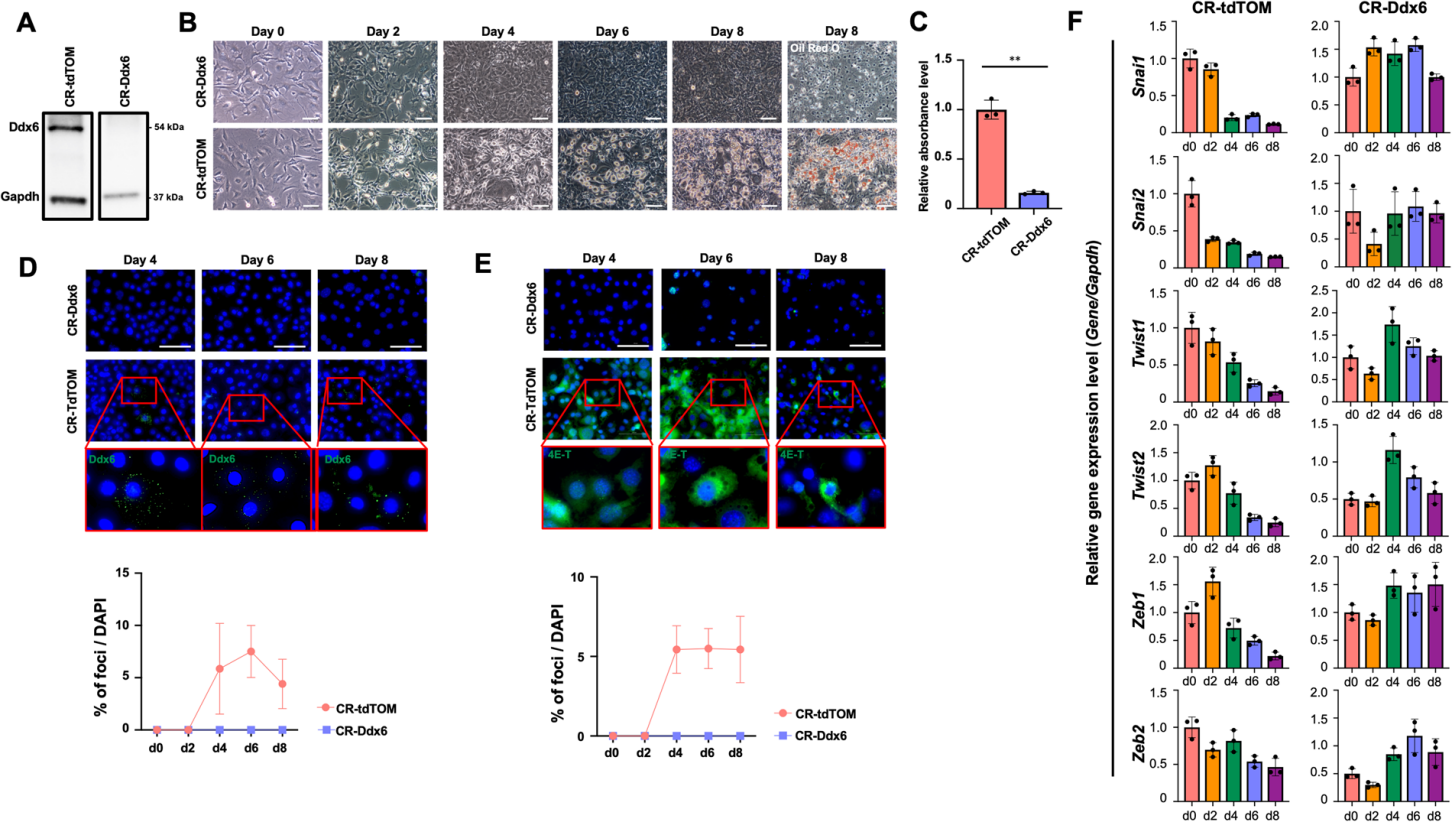
Adipogenic differentiation of 3T3L1 Ddx6-KO preadipocytes. **A** Western blotting of 3T3L1 Ddx6-KO preadipocytes. **B** Phase-contrast microscopic images during adipogenesis. On day 8, cells were fixed and stained with oil red O. The scale bar is 100 μm. **C** Relative absorbance of oil red O. The error bars indicate the SDs (*n* = 3). Double asterisks (**) indicate significance (*P* < 0.01). **D** Immunocytochemistry of Ddx6 foci and the proportion of cells with foci during adipogenesis. The scale bar is 50 μm. The error bars indicate the SDs (*n* = 6). **E** Immunocytochemistry of 4E-T foci and the proportion of cells with foci during adipogenesis. The scale bar is 50 μm. The error bars indicate the SDs (*n* = 6). **F** RT-qPCR analysis of gene expression during adipogenesis. Individual RNA expression levels were normalized to Gapdh expression levels. The error bars indicate the SDs (*n* = 3).

Ddx6 was knocked out in 3T3L1 cells to generate 3T3L1 Ddx6-KO cells by introducing a recombinant lentivirus carrying Cas9 and guide RNA (gRNA) to the Ddx6 sequence from sites 44,614,295 to 44,614,314 on chromosome 9 (Fig. 2A). The 3T3L1 Ddx6-KO cells and mock-transfected (3T3L1 tdTomato [TOM]) cells were subjected to adipogenic differentiation. The 3T3L1 Ddx6-KO cells clearly failed to differentiate into adipocytes according to macroscopic observation, whereas the mock-transfected cells differentiated similarly to the native cells (Fig. 2B). Oil red O staining demonstrated the absence of lipid droplets in 3T3L1 Ddx6-KO cells, which had a negligible level of the signal; in contrast, mock transfectants had positive red staining in the culture dish with a positive signal similar to that of native 3T3L1 cells (Fig. 2B, C). DDX6 was knocked out in human mesenchymal stem cells (hMSCs) in the same way as in 3T3L1 cells (Supplementary Fig. S3). Among hMSC Ddx6-KO cells subjected to an adipogenesis induction protocol, significant suppression of adipocyte differentiation was observed via macroscopic analysis and verified by oil red O staining (Supplementary Fig. S4). 4E-T is an essential component of PBs, similar to Ddx6; thus, 4E-T staining was selected as an alternative method of identification of PBs in Ddx6-KO experiments. The numbers of PBs generated in the mock transfectants were similar when Ddx6 staining and 4E-T staining were used for detection; however, 3T3L1 Ddx6-KO cells did not form PBs (Fig. 2D, E). In addition, the mesenchymal transcription factors Snail 1/2, Twist 1/2, and Zeb 1/2 were maintained at constant levels during adipogenesis in 3T3L1 Ddx6-KO cells (Fig. 2F). The mock transfectants had the same kinetics of gene expression of these transcription factors as that observed in the native 3T3L1 cells, indicating that recombinant lentiviral transduction did not influence differentiation. These results indicate that Ddx6 KO is directly linked to a lack of PB generation and failure of adipogenesis due to maintenance of the parental transcriptome.

### Inhibition of adipogenesis by 4E-T knockdown (KD)

**Fig. 3:**
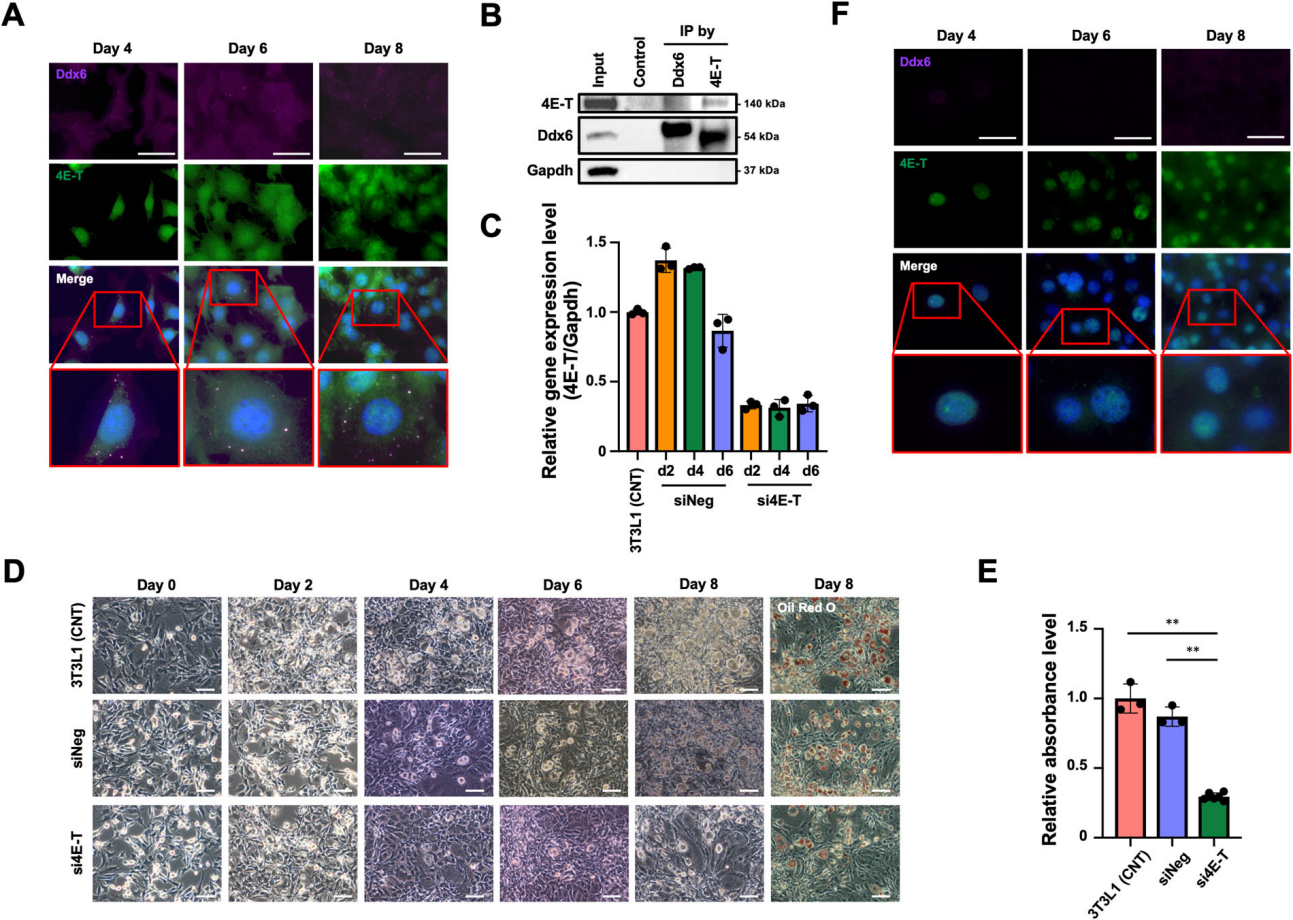
Adipogenic differentiation of 3T3L1 4E-T-KD preadipocytes. **A** Immunocytochemistry of 3T3L1 preadipocytes during adipogenesis. Foci of Ddx6 and 4E-T proteins were merged. The scale bar is 50 μm. Nuclei were stained with DAPI. **B** Immunoprecipitates bound by Ddx6 or 4E-T antibodies were analyzed by western blotting with anti-Ddx6 and anti-4E-T antibodies. **C** RT-qPCR analysis of 4E-T expression on each day. Individual RNA expression levels were normalized to Gapdh expression levels. The error bars indicate the SDs (*n* = 3). **D** Phase-contrast microscopy images during adipogenesis. On day 8, the cells were fixed and stained with oil red O. The scale bar is 100 μm. **E** Relative absorbance of oil red O. The error bars indicate the SDs (*n* = 3). Double asterisks (**) indicate signific ance (*P* < 0.01). **F** Immunocytochemistry of 3T3L1 4E-T-KD preadipocytes during adipogenesis. The scale bar is 50 μm. The nuclei were stained with DAPI.

Ddx6 functions as a driver of RNA decay and translational repression and participates in nuclear export and translational activation^25^; thus, KD of 4E-T in 3T3L1 cells was performed to determine if this binding partner of Ddx6 in PBs is involved in inhibition of adipogenesis and to identify the role of PB-dependent RNA metabolism in adipogenesis. Colocalization of Ddx6 and 4E-T in distinctive foci in the cytoplasm of 3T3L1 cells was detected from day 4 to day 8 of adipogenic induction (Fig. 3A). Immunoprecipitation analysis using anti-Ddx6 and anti-4E-T antibodies in 3T3L1 cells on day 4 of adipogenic induction clearly demonstrated the presence of 4E-T and Ddx6, respectively (Fig. 3B). To KD 4E-T expression, three independent siRNAs were designed to exclude nonspecific off-target effects. Two types of siRNAs against 4E-T suppressed 4E-T expression to less than 50% of baseline levels for at least 5 days (Supplementary Fig. S1). Transfection of 3T3L1 cells with siRNA against 4E-T was performed 1 day prior to adipogenic induction, and the suppression of 4E-T expression was maintained for 4 days without differentiation. The resulting cells were designated 3T3L1 4E-T-KD cells (Fig. 3C). Adipogenic induction in 3T3L1 4E-T-KD cells was suppressed, as indicated by the low number of lipid-laden adipocytes (Fig. 3D) and by the levels of eluted Oil Red (Fig. 3E). 3T3L1 4E-T-KD cells were stained with anti-Ddx6 antibodies to identify PBs during adipogenesis. The ~50% reduction in the level of 4E-T expression prevented the formation of PBs during adipogenic induction, similar to the situation in 3T3L1 Ddx6-KO cells (Fig. 3F). Inhibition of adipogenesis in 3T3L1 4E-T-KD cells was incomplete, while it was almost complete in 3T3L1 Ddx6-KO cells; the differences between KO and KD could have influenced the outcome. These results suggest that a functional overlap of Ddx6 and 4E-T in the context of adipogenic induction signals involves RNA decay or translational repression by PBs, which may be indispensable for adipogenesis.

### Overexpression of DDX6 does not influence adipogenesis

**Fig. 4:**
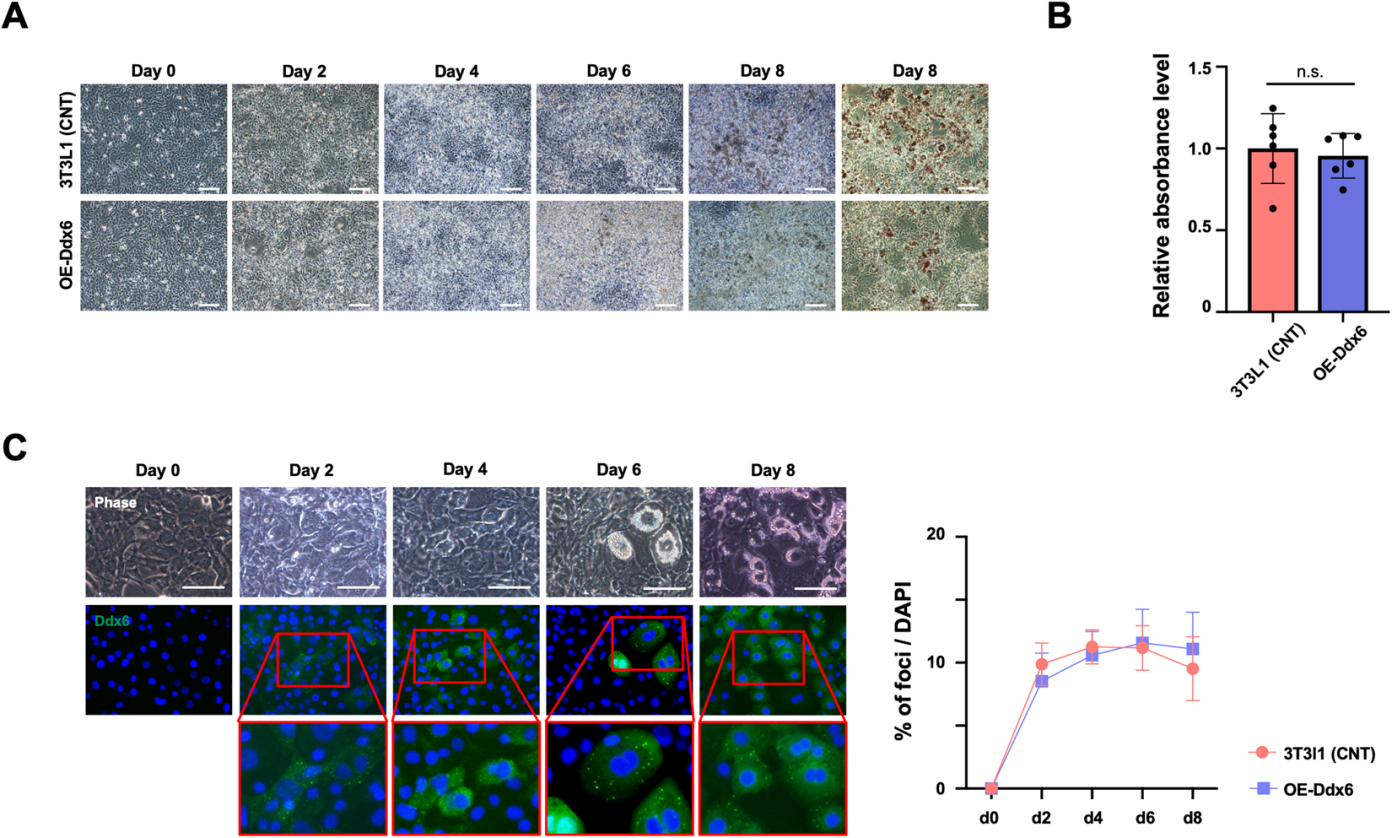
Adipogenic differentiation of Ddx6-O/E 3T3L1 preadipocytes. **A** Phase-contrast microscopic images during adipogenesis. On day 8, the cells were fixed and stained with oil red O. The scale bar is 100 μm. **B** Relative absorbance of oil red O. The error bars indicate the SDs (*n* = 6). **C** Fluorescence microscopy images and the proportion of cells with foci during adipogenesis are shown. The scale bar is 100 μm. The error bars indicate the SDs (*n* = 6). The nuclei were stained with Hoechst 33342.

Then, the effects of overexpression of Ddx6 in 3T3L1 cells on adipogenesis were tested. Constitutively, Ddx6-overexpressing (O/E) 3T3L1 cells were created by transfection with a recombinant retrovirus carrying Ddx6 and subsequent selection (Supplementary Fig. S2). 3T3L1 Ddx6 O/E cells were subjected to the adipogenic induction protocol and demonstrated macroscopic characteristics similar to those of native 3T3L1 cells during the process (Fig. 4A). Oil red O staining demonstrated similar numbers of lipid droplets in 3T3L1 Ddx6 O/E and native 3T3L1 cells, and the levels of staining were not significantly different between these cell types (Fig. 4B). PBs were identified by fluorescence microscopy, and the numbers of PBs were similar in 3T3L1 Ddx6 O/E and native cells (Fig. 4C). The level of DDX6 in the cytoplasm has been reported to be as high as 3.3 μM in HeLa cells^26^, indicating that RNA metabolism-dependent regulation of the transcriptome may be able to respond rapidly to environmental cues. Apparently, overexpression of Ddx6 does not influence physiological RNA metabolism.

### Transcriptome analysis in 3T3L1 Ddx6-KO cells

**Fig. 5:**
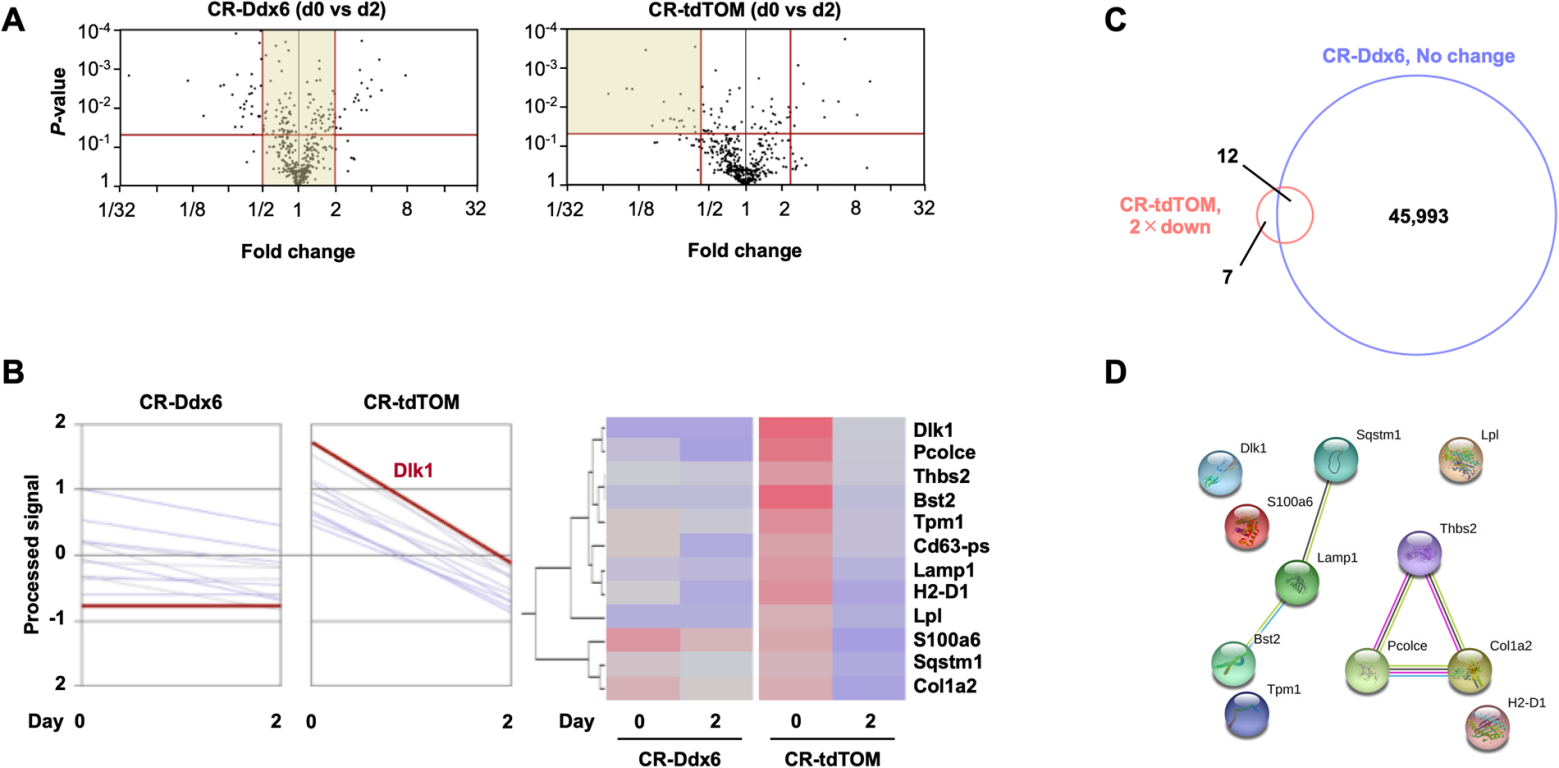
Expression profiling of mRNA changes during the early phase of adipogenic differentiation. **A** Volcano plots at the early phase of adipogenesis in TOM-transfected and 3T3L1 Ddx6-KD preadipocytes. **B** Heatmap of 12 selected genes whose expression was reduced twofold in 3T3L1 TOM cells but not in 3T3L1 Ddx6-KD cells. **C** Venn diagram showing the number of mRNAs relevant to each segment. **D** Protein–protein interaction network of 12 intersecting genes predicted by STRING analysis.

Ddx6 and 4E-T are known to be important factors in PB formation and to play important roles in RNA metabolism^27^. These factors are involved in RNA degradation in PBs; hence, we hypothesized that degradation of mRNAs specifically expressed in cells before induction is essential for the induction of adipogenesis. To test this hypothesis, a global gene expression analysis was performed in 3T3L1 Ddx6-KO cells before and after adipogenic induction using RNA-seq. The analysis focused on a group of genes whose expression was reduced twofold in mock transfectants but was not reduced in 3T3L1 Ddx6-KO cells (Fig. 5A, B). The results showed that the levels of 19 genes were reduced by twofold and that the levels of 46,005 genes were unchanged in 3T3L1 Ddx6-KO cells. These results were evaluated using a Venn diagram, and 12 genes were identified as consistently downregulated genes (Fig. 5C). The STRING database (https://string-db.org) was used to analyze the relationships among the 12 corresponding proteins. Two groups included Sqstm1 and Bst2 centered on Lamp1, and thrombospondin 2 (Thbs2), Pcolce, and Col1a2 were related to each other (Fig. 5D). Thbs2 has been reported to have weak suppressive activity on adipogenesis^28^, and the expression of Col1a2 and Pcolce, two other extracellular matrix proteins, decreases as a result of adipogenesis progression^29^, suggesting that these molecules are not crucial negative regulators. Another gene set related to autophagy has a link to RNA metabolism^30^. However, these proteins are less characterized than Dlk1 in the context of adipogenesis^31^. Therefore, subsequent experiments were focused on Dlk1 (Pref1), a negative regulator of adipogenesis.

### Dlk1 KO in 3T3L1 Ddx6-KO cells does not rescue adipogenesis

**Fig. 6:**
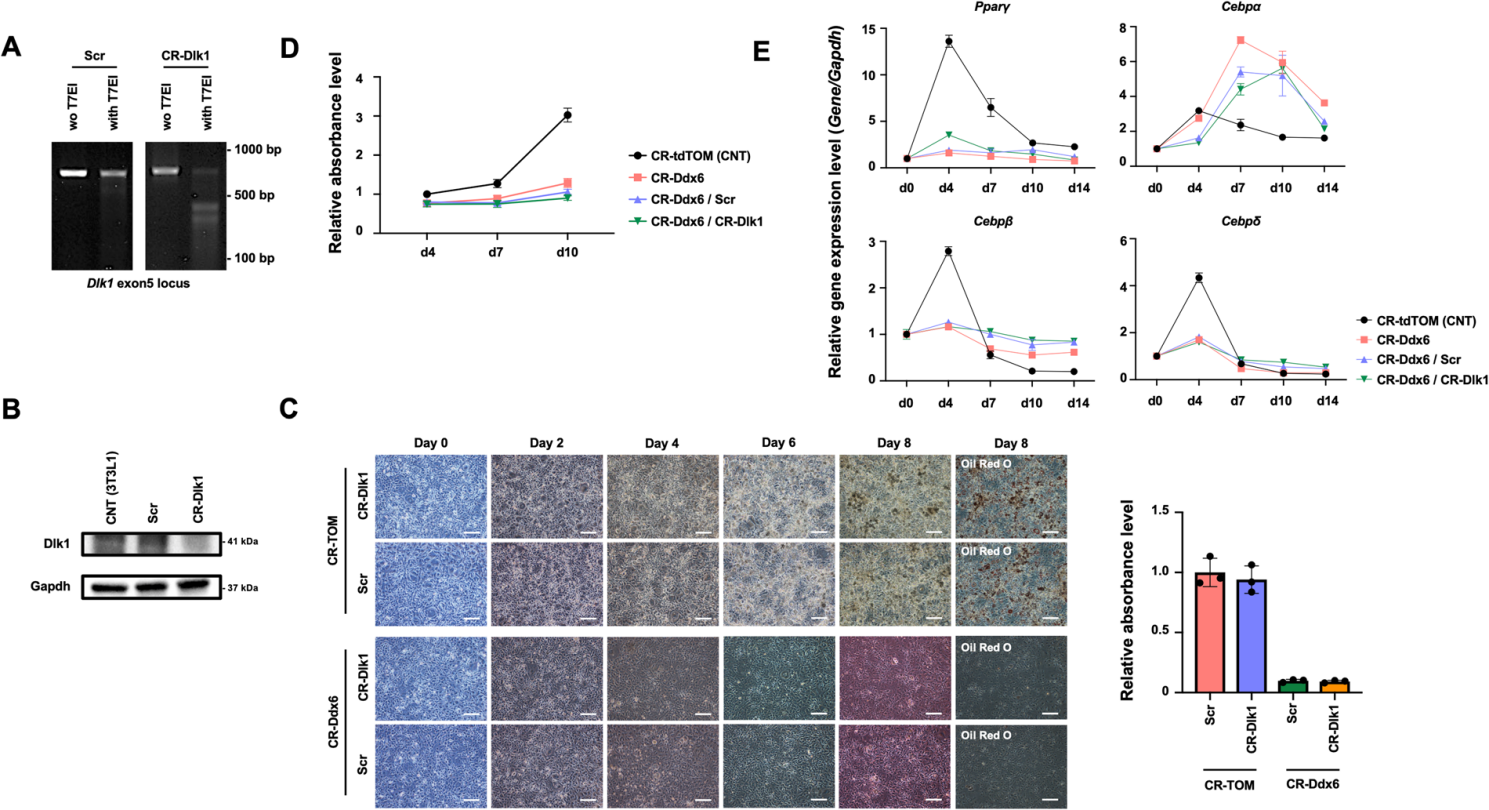
Adipogenic differentiation of 3T3L1 Dlk1-KO preadipocytes. **A** T7EI mismatch detection assay for assessment of the efficiency of the CRISPR-Cas9 system. **B** Western blot verification of Dlk1 KO by the CRISPR-Cas9 system. **C** Phase-contrast microscopy images during adipogenesis and the relative absorbance of oil red O on day 8. The scale bar is 100 μm. The error bars indicate the SDs (*n* = 3). **D** Relative absorbance of oil red O on each day. The error bars indicate the SDs (*n* = 3). **E** RT-qPCR analysis of the expression of each gene during adipogenesis. Individual RNA expression levels were normalized to Gapdh expression levels. The error bars indicate the SDs (*n* = 3).

The expression of the gene Dlk1 is significantly reduced by Ddx6-mediated RNA decay, and Dlk1 has been characterized as a negative regulator of adipogenesis^32^. 3T3L1 Ddx6-KO cells maintained Dlk1 expression during adipogenic induction (Fig. 5B). Dlk1 was knocked out by lentiviral transfection of clustered regularly interspaced short palindromic repeats (CRISPR)-Cas9 and gRNA in 3T3L1 Ddx6-KO cells; the resulting cells were designated 3T3L1 Ddx6/Dlk1-double KO (dKO) cells (Fig. 6A, B). dKO was used to determine whether persistent Dlk1 expression is a major cause of the antiadipogenic effects in 3T3L1 Ddx6-KO cells. 3T3L1 Ddx6-KO cells and 3T3L1 mock transfectants were genetically modified with lentiviruses carrying gRNA for Dlk1 KO or scrambled gRNA; the corresponding cells were designated 3T3L1 Ddx6/Dlk1-dKO, 3T3L1 Ddx6-KO/Scramble, 3T3L1 TOM/Dlk1 KO, and 3T3L1 TOM/Scramble cells. All genetically modified cells were subjected to adipogenic induction. In 3T3L1 Ddx6/Dlk1-dKO cells, lipid-laden cells were macroscopically rare; these observations were verified by oil red O staining and quantification. A similar pattern was observed in 3T3L1 Ddx6-KO cells (Fig. 6C, D), suggesting that the inhibitory effect of Ddx6 KO on adipogenesis is attributable to the persistence of an array of transcripts in parental cells, not only Dlk1 transcripts. In contrast, 3T3L1 TOM/Dlk1 KO and 3T3L1 TOM/Scramble cells had numerous lipid-laden adipocytes, and the kinetics were similar to those in native 3T3L1 cells; these data confirm that the transfection procedures did not influence the adipogenicity of 3T3L1 cells. In 3T3L1 Ddx6/Dlk1-dKO, 3T3L1 Ddx6-KO/Scramble, and 3T3L1 Ddx6-KO cells, major regulators of adipogenesis, including C/EBPβ, C/EBPδ, and PPARγ, were initially expressed during adipogenic induction, whereas in 3T3L1 TOM cells, the levels of these genes increased during adipogenic induction in a pattern similar to that observed in native 3T3L1 cells (Fig. 6E). Another major regulator, C/EBPα, demonstrated greater increases in 3T3L1 Ddx6/Dlk1-dKO, 3T3L1 Ddx6-KO/Scramble, and 3T3L1 Ddx6-KO cells than in 3T3L1 TOM cells in the late phase of adipogenesis. The inability of Ddx6-defective cells to proceed to adipogenic differentiation despite enhanced C/EBPα levels indicated that the Ddx6-dependent RNA decay machinery is indispensable for adipogenesis.

## Discussion

**Fig. 7:**
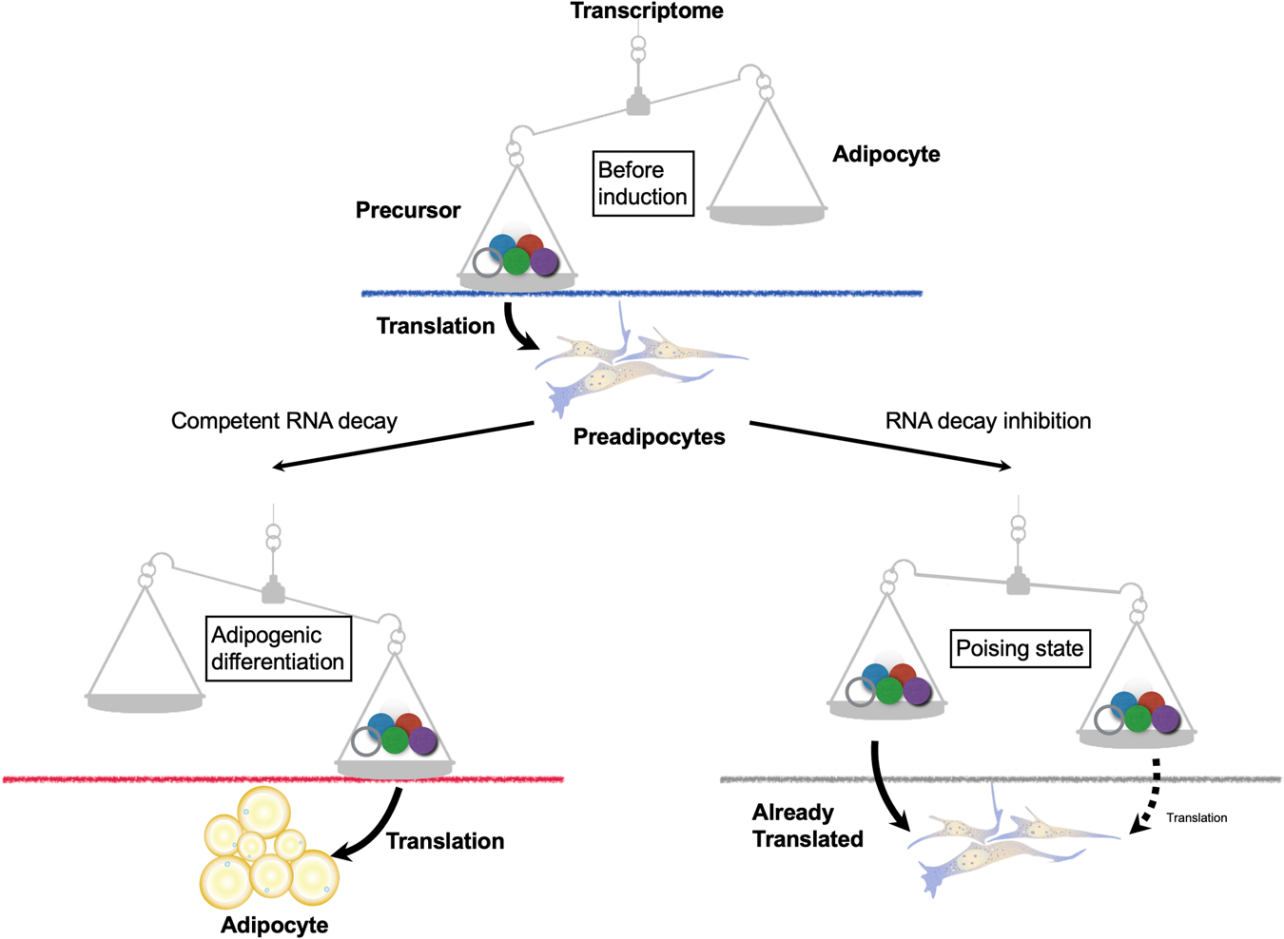
Model of adipogenic differentiation relevant to RNA metabolism.

The results of this study indicate that clearance of the transcripts shaping the phenotype of preadipocytes is indispensable for adipogenesis, the transformation of preadipocytes to lipid-laden adipocytes. The molecular machinery used to erase mRNAs of the parental cells includes DDX6 and 4E-T; these molecules work together in the PBs, which are membraneless organelles. A model of adipogenic differentiation with consideration of RNA metabolism is presented in Fig. 7. Inhibition of RNA decay may overload the translational machinery by preserving old transcripts for the precursors, thus preventing efficient translation of the nascent transcripts for adipocytes. On the other hand, complete RNA decay releases the translational machinery, which leads to translation of nascent transcripts and consequentially to maturation of adipocytes. Consistent with the findings of our previous study that reprogramming to pluripotency requires RNA decay^8^, the results of this study suggest that cellular transition can actively remove the transcriptome of the parental phenotype via RNA decay to generate a new transcriptome of the transformed phenotype.

Transcriptional regulation by transcription factors and epigenetic modifiers has been investigated in detail in the context of adipogenesis; however, posttranscriptional mechanisms, such as RNA metabolism, require additional study. A comprehensive analysis of the transcriptional and epigenomic changes during adipogenesis of mesenchymal stem cells has demonstrated significant remodeling of the chromatin landscape and de novo activation of enhancers^33^. Comprehensive analyses have also revealed that cells differentiating into adipocytes eliminate the stem cell-related transcriptome and activate a phenotypic transcriptome. Murine embryonic stem cells deficient in SMG6, which is a key regulator of nonsense-mediated mRNA decay, are unable to differentiate into all three germ layers^34^. Conversely, maintenance of stemness in epidermal progenitor cells requires that mRNAs that promote differentiation programs, such as KLF4, are targeted for degradation by Ddx6-related machinery via GC-rich regions in their 5′-untranslated regions^35^. Although there are a limited number of reports that RNA decay plays a crucial role in posttranscriptional regulation to maintain homeostasis in a context-dependent manner, this study is the first to demonstrate that RNA decay impacts adipogenesis.

Adipocytes play an essential role in the efficient storage of energy in the form of triglycerides. However, accumulation of lipid intermediates in hepatocytes leads to cellular dysfunction and insulin resistance, which is known as lipotoxicity, resulting in nonalcoholic fatty liver disease. This disease can develop into more serious conditions, including nonalcoholic steatohepatitis; a similar process in cardiomyocytes may result in heart failure due to apoptotic cell death^36^. Upon overnutrition, adipocytes can adapt via hyperplasia, an increase in the number of adipocytes, or via hypertrophy, an increase in the size of adipocytes. In adults, overnutrition is likely to lead to accumulation of adipose tissue containing hypertrophied adipocytes. In hypertrophied adipose tissues, maladaptation results in hypovascularity due to an insufficient proangiogenic effect of hypoxia-inducible factor 1^37^; thus, the tissue becomes susceptible to damage induced by subsequent increases in the release of proinflammatory and profibrotic factors^38^. This phenomenon is linked to necrosis, immune cell infiltration, and fibrosis^39^. On the other hand, a body of evidence indicates that hyperplastic expansion of adipose tissue in obesity and metabolic syndrome is involved in maintenance of metabolic health in animal models overexpressing adipogenesis-promoting genes^40,41^. Drugs of the thiazolidinedione (TZD) class are potent PPARγ ligands used to treat diabetes mellitus that stimulate adipogenesis to induce the hyperplastic adaptation of adipocytes and improve metabolism^42^. Dysfunction of RNA decay may participate in hypertrophy of adipocytes by suppressing adipogenesis.

Systemic induction of adipogenesis to maintain metabolic health under conditions of overnutrition can involve ectopic adipogenesis in the bone marrow, skeletal muscles, liver, and heart. Hematopoietic tissues are replaced with adipocytes under various conditions, such as obesity, aging, and administration of TZDs^43^. Wnt/β-catenin signaling is indispensable for the differentiation of mesenchymal precursor cells to osteocytes, and loss of the signal results in bone marrow adiposity^44^. During hematopoietic stem cell (HSC) transplantation following total-body irradiation, a PPARγ inhibitor has been found to ameliorate the engraftment of donor HSCs and support hematopoietic reconstruction^45^. In addition, repair of fractured bone is postponed by adipocyte accumulation in the bone marrow, resulting in bone frailty in humans^36^. Adipocyte infiltration in skeletal muscle during injury repair deteriorates function and is linked to sarcopenia in humans; this effect is suppressed by blockade of Hedgehog signaling, which restricts adipogenesis via TIMP3 and supports muscle regeneration^46^. In the heart after myocardial infarction, fibrofatty tissue replacement in scarred regions has been documented^47,48^, and adipocyte deposits can cause lethal arrhythmia^49^. Ectopic accumulation of fat tissue is a potential clinical target for the suppression of adipogenesis that currently does not have any drug treatment options. Modulation of this RNA decay pathway could offer novel pharmacological strategy for the abovementioned disorders.

Arrhythmogenic cardiomyopathy, which was originally referred to as arrhythmogenic right ventricular dysplasia^50^/cardiomyopathy^51^, is a genetic disorder characterized by dilated ventricular remodeling with fibrofatty replacement of the myocardium with classical right ventricular and rare left ventricular or biventricular phenotypes^52^. This cardiac adipose replacement process is attributed to adipogenesis, which forms adipose tissues, and does not involve lipogenesis in cardiomyocytes, which generates intracellular lipid droplets; however, the origins of the adipocytes remain controversial and may include cardiomyocyte transdifferentiation^53^, cardiac progenitor cells^54^, and pluripotent cells^55^. The inducers of this phenomenon include genes encoding the components of desmosomes, such as plakoglobin (also known as γ-catenin), which competes with β-catenin in wnt signaling^56^; cytoskeletal components, such as lamin a/c^57^; and transforming growth factor β^58^. The initial commitment to adipogenic differentiation in arrhythmogenic cardiomyopathy might be different from that in normal adipogenesis, and an inducer, such as endurance exercise, may be required for the development of a clinical phenotype^59^; however, adipocyte maturation during adipogenesis in adipose tissue and in the myocardium in the context of cardiomyopathy may share the molecular machinery^60^. An intervention to suppress adipogenesis in arrhythmogenic cardiomyopathy may be a potential novel treatment for intractable diseases.

This study investigated the involvement of RNA decay during cellular transition; the results indicate that the molecular machinery of RNA decay may be a therapeutic target for ectopic fat deposition. RNA decay includes various mechanisms, such as ARE-mediated, GU-mediated, and CDE-mediated mRNA decay mechanisms that depend on target sequences^61^; thus, additional studies on RNA decay in adipogenesis may lead to the development of a drug that can be used to treat ectopic adipocyte accumulation.

## Materials and methods

### Cell culture and adipocyte differentiation

3T3L1 preadipocytes that were purchased from Japanese Collection of Research Bioresources Cell Bank (#JCRB9014, Japanese Collection of Research Bioresources Cell Bank, Osaka, Japan) at the beginning of this study were cultured in high-glucose Dulbecco’s modified Eagle’s medium (DMEM, #043-30085, Fujifilm Wako Pure Chemical, Osaka, Japan) supplemented with 10% fetal bovine serum (FBS, #16000-044, Life Technologies, Carlsbad, CA, USA) and 1% penicillin/streptomycin (#15070063, Thermo Fisher Scientific Inc., Waltham, MA, USA) and incubated at 37 °C in a humidified 5% CO_2_ incubator. We have verified each several months, all cultured cells are negative for mycoplasma infection. For adipocyte differentiation, the cells were seeded at 2 × 10^5^ per well in six-well cell culture plates (#353046, Corning Inc., Corning, NY, USA) in growth medium (day −1). On the next day (day 0), the cells were maintained in high-glucose DMEM supplemented with 10% FBS, 1% penicillin/streptomycin, and an adipogenic differentiation cocktail containing 1 μM dexamethasone (#D1756, Sigma-Aldrich Corp., Saint Louis, MO, USA), 0.5 mM IBMX (#I5879, Sigma-Aldrich), and 5 μg/ml insulin (#093-06471, Fujifilm Wako). After exposure to the adipogenic differentiation cocktail, the cells were maintained in fresh high-glucose DMEM supplemented with 10% FBS, 1% penicillin/streptomycin, and insulin (5 μg/ml), and the medium was changed every other day.

hMSCs that researchers can use without permission and deliberation by ethical committee were obtained from Lonza (#PT-2501, Lonza, Walkersville, MD, USA). The hMSCs were cultured in MSCBM basal medium supplemented with the components of an MSCGM SingleQuots Supplement Kit (#PT-3001, Lonza, Walkersville, MD, USA) and incubated at 37 °C in a humidified 5% CO2 incubator. For adipocyte differentiation, the cells were seeded at 2 × 10^5^ per well in six-well cell culture plates (Corning Inc.) in growth medium. The next day, the cells were switched to hMSC adipogenic induction medium (#PT-3004, Lonza) and cultured for 3 days. The cells were then cultured for 1 day in supplemented hMSC adipogenic maintenance medium (#PT-3004, Lonza) according to the manufacturer’s instructions. Three cycles of culture in induction and maintenance medium were performed to stimulate optimal adipogenic differentiation.

### Overexpression of Ddx6

For overexpression of Ddx6 in 3T3L1 preadipocytes, the sequence of Ddx6 fused with EGFP was inserted into the retroviral vector pMXs containing a puromycin resistance gene (#RTV-012, Cell Biolabs Inc., San Diego, CA, USA) (Supplementary Fig. 2). The pMXs retroviral vector carrying Ddx6 with EGFP was transfected into 3T3L1 preadipocytes, and transfected cells were selected in the presence of puromycin.

### Lentiviral transduction for Ddx6 KO in 3T3L1 preadipocytes

The Ddx6 gene was knocked out using CRISPR-Cas9 technology. The Ddx6 gene was knocked out using CRISPR-Cas9 technology. sgRNA targeting TOM was used as a negative control and was cloned together with Ddx6 exon 4 into a Cas9-expressing lentiviral transfer vector (lentiCRISPRv2, Addgene #52961, Addgene, Watertown, MA, USA) according to the method described by Feng Zhang. The following sense oligonucleotides were used for the gRNAs targeting TOM and Ddx6 exon 4: 5′-CACCGCCCCGCGACGGCGTGCTGAA-3′ (forward)/5′-AAACTTCAGCACGCCGTCGCGGGGC-3′ (reverse) and 5′-CACCGGGAAAAACCATCTCCTATCC-3′ (forward)/5′-AAACGGATAGGAGATGGTTTTTCCC-3′ (reverse), respectively. These oligonucleotides were ligated into lentiCRISPRv2 according to the lentiCRISPRv2 and lentiGuide oligo cloning protocol of Feng Zhang. To prepare lentiviruses for TOM and Ddx6 gene disruption, lentiCRISPRv2–sgRNA TOM and Ddx6 transfer plasmids were cotransfected with the packaging plasmids pMD2.G and psPAX2 (Addgene plasmids 12259 and 12260, respectively). For viral transduction for gene disruption of TOM and Ddx6, 1 × 10^5^ 3T3L1 preadipocytes were incubated with 0.2 μm-filtered lentivirus-containing supernatant. Three days after infection, puromycin was added to select sgRNA/Cas9-positive cells. After puromycin selection, the cells were diluted into a single-cell suspension by limited dilution for single-cell cloning. To assess the efficiency of sgRNA-guided Cas9 cutting in the Ddx6 genomic sequence, the protein deletions were confirmed by western blotting.

### Lentiviral transduction for DDX6 KO in hMSCs

To KO the DDX6 gene using CRISPR-Cas9 technology as mentioned above, we designed plasmids and generated transfectants with them as described in our previous report^8^. Briefly, the sgRNAs targeting DDX6 exon 1 and a scramble sequence were cloned into a Cas9-expressing lentiviral transfer vector (Addgene). The following oligonucleotides were used for the DDX6 exon 1-targeting gRNA and the scramble sequence: 5′-CACCGTATAACAGGGTTCTCTGTTC-3′ (forward)/5′-AAACGAACAGAGAACCCTGTTATAC-3′ (reverse) and 5′-CACCGGCACTCACATCGCTACATCA-3′ (forward)/5′-AAACTGATGTAGCGATGTGAGTGCC-3′ (reverse), respectively. These oligonucleotides were ligated into lentiCRISPRv2. The lentiCRISPRv2–sgRNA DDX6 and scramble transfer plasmids were cotransfected with the packaging plasmids pMD2.G and psPAX2 (Addgene). For viral transduction-mediated DDX6 disruption and transduction of the scramble sequence, 1 × 10^5^ hMSCs were incubated with 0.2 μm-filtered lentivirus-containing supernatant. Three days after infection, puromycin was added to select sgRNA/Cas9-positive cells. After puromycin selection, the protein deletions were confirmed by western blotting to assess the efficiency of sgRNA-guided Cas9 cutting in the DDX6 genomic sequence.

### 4E-T silencing using siRNA transfection

Validated mouse 4E-T siRNA #1 (siRNA ID: s92476, antisense region located at exon 4 of 4E-T), 4E-T siRNA #2 (siRNA ID: s92477, antisense region located at exon 6 of 4E-T), and a nontargeting siRNA (#AM4611) as the negative control were obtained from a Silencer Select Predesigned siRNA Library (Thermo Fisher Scientific Inc.). According to the manufacturer’s instructions, 3T3L1 preadipocytes were seeded at a density of 2 × 10^5^ cells per well in six-well plates in 2 ml of high-glucose DMEM with 10% FBS and 1% penicillin/streptomycin. After overnight culture, siRNAs at a final concentration of 5 nM were premixed with 9 μl RNAiMAX transfection reagent (#13778075, Thermo Fisher Scientific Inc.) in 300 μl of Opti-MEM (#31985070, Thermo Fisher Scientific Inc.). Then, the mixture was added into each well. Eighteen hours after transfection, the medium containing siRNA was replaced with fresh medium with adipogenic differentiation cocktail to induce differentiation. Four days after transfection, the siRNA mixture was added again as described above to maintain the effect of 4E-T silencing.

### Dlk1 KO using the Alt-R CRISPR-Cas9 system

To KO Dlk1, validated mouse Dlk1 Alt-R CRISPR-Cas9 gRNA (crRNA) #1 (Design ID: Mm.Cas9.DLK1.1. AA), Dlk1 crRNA #2 (Design ID: Mm.Cas9.DLK1.1. AB), Dlk1 crRNA #3 (Design ID: Mm.Cas9.DLK1.1. AC) and Alt-R S.p. Cas9 nuclease V3 (catalog number: #1081058) as the negative control were obtained from the Alt-R CRISPR-Cas9 System (Integrated DNA Technologies Inc., Coralville, IA, USA). According to the manufacturer’s instructions, crRNAs at a final concentration of 10 nM were premixed with the same amount of tracrRNA (#1072532, Integrated DNA Technologies Inc.) in 1× nuclease-free duplex buffer (Integrated DNA Technologies Inc.). Then, the mixture was mixed with Alt-R S.p. Cas9 nuclease (#1081058, Integrated DNA Technologies Inc.) in Opti-MEM (Thermo Fisher Scientific Inc.) to form the RNP complex. The RNP complex was mixed with RNAiMAX transfection reagent (Thermo Fisher Scientific Inc.) in Opti-MEM. Then, the final mixture was added to wells containing 3T3L1 preadipocytes at 1 × 10^5^ per well in six-well plates in 2 ml of high-glucose DMEM with 10% FBS without penicillin/streptomycin. Eighteen hours after transfection, the medium containing the RNP complex was replaced with fresh medium with adipogenic differentiation cocktail to induce differentiation. The efficiency of Dlk1 gene disruption was confirmed by T7 endonuclease I (T7EI) assay.

### T7EI mismatch detection assay

To evaluate the efficiency of Dlk1 gene disruption, a T7EI (#M0302, New England Biolabs Inc., Ipswich, MA, USA) mismatch detection assay was used. The genomic DNA from Dlk1-KO cells treated with CRISPR-Cas9 reagents was amplified by PCR to ensure that the amplicon covered the site of the CRISPR gRNA target. PCR was performed with GoTaq Green Master Mix (#M7122, Promega Corporation, Madison, WI, USA) on a T100 thermal cycler (Bio-Rad Laboratories Inc. Hercules, CA, USA)) according to the manufacturer’s instructions. The following sets of primers designed for the CRISPR gRNA target site were used: oligonucleotide set 1: 5′-GGACGTGGGAGGTCGTTTC-3′ (forward) and 5′-TTCTTGCGAAGCATGTGGTTG-3′ (reverse); and oligonucleotide set 2: 5′-TCTCACCGATGGCCTTCCTA-3′ (forward) and 5′-CACCTCCACCCCCATTTCAA-3′ (reverse). Heteroduplex formation was performed using NEB buffer 2 (#B7002, New England Biolabs Inc.) on a T100 thermal cycler (Bio-Rad Laboratories Inc.) according to the manufacturer’s instructions. The heteroduplexes were digested with T7EI (New England Biolabs Inc.) at 37 °C for 20 min, and the products were electrophoresed through a 2% agarose gel. The bands were visualized using a VersaDoc system (Bio-Rad Laboratories Inc., Hercules, CA, USA).

### Total RNA extraction and reverse transcription-quantitative polymerase chain reaction (RT-qPCR) analysis

Total RNA from the cells was extracted using TRIzol (#15596018, Life Technologies, Carlsbad, CA, USA) and a Direct-zol RNA MiniPrep Kit (#R2052, Zymo Research, Irvine, CA, USA) with DNase I according to the manufacturer’s recommendations. To perform the RT-qPCR assay, 400 ng of total RNA was reverse-transcribed using a PrimeScript RT Reagent Kit (#RR037, Takara Bio, Shiga, Japan) and a T100 thermal cycler (Bio-Rad Laboratories Inc.). RT-qPCR was performed with KAPA SYBR FAST qPCR Kit Universal Master Mix (2×) (#7959397001, Kapa Biosystems Ltd., Wilmington, MA, USA) on a CFX Connect Real-Time System (Bio-Rad Laboratories Inc.). The relative gene expression levels were normalized to Gapdh expression.

### Oil red O staining and quantification

Fifteen micrograms of oil red O powder (#1320-06-5, Sigma-Aldrich) was dissolved in 30 ml of 100% isopropyl alcohol by gentle heating in a water bath at 37 °C. After dissolution, the solution was diluted with 20 ml of distilled water and filtered to remove undissolved powder. Cells in cell culture plates were washed with phosphate-buffered saline (PBS, #166-23555, Fujifilm Wako) twice, fixed with 4% paraformaldehyde (PFA, #163-20145, Fujifilm Wako) for 10 min at room temperature, and rinsed with 60% isopropyl alcohol. The samples were stained with oil red O solution for 30 min at room temperature. After fat droplets in adipocytes were stained, the samples were rinsed with 60% isopropyl alcohol again. Oil red O-stained cells were observed, and images were captured with an IX71 inverted microscope (Olympus, Tokyo, Japan). For quantification of adipogenesis, oil red O was dissolved in 100% isopropyl alcohol. The absorbance of each sample was measured by an iMark microplate reader (Bio-Rad Laboratories Inc.) at 492 nm.

### Western blotting

Cytoplasmic protein (50 μg) was dissolved in RIPA buffer (#182-02451, Fujifilm Wako), boiled for 10 min, electrophoresed through a 10% SDS polyacrylamide gel, and electroblotted onto a PVDF transfer membrane (#IPVH00010, Millipore, Billerica, MA, USA). The membrane was blocked with PBS containing 5% skim milk and 0.05% Tween 20 and incubated for 1 h with a Ddx6 antibody (#GTX102795, GeneTex Inc., Irvine, CA, USA) diluted 1:500 with blocking buffer. After washing, the membrane was incubated with a 1:5000 dilution of horseradish peroxidase-linked goat anti-rabbit IgG (#7074, Cell Signaling Technology, Inc., Danvers, Massachusetts, USA) in blocking buffer. Subsequently, the blots were developed using an enhanced chemiluminescence detection kit substrate (#1705060, Bio-Rad Laboratories Inc.), and the protein bands were visualized using a VersaDoc system (Bio-Rad Laboratories Inc.).

### Immunocytochemistry

Cells were fixed with 4% PFA at 4 °C for 5 min and permeabilized with 0.1% Triton X-100 at room temperature for 20 min in the presence of a protein-blocking solution consisting of PBS supplemented with 5% normal goat serum (#X090710-8, Agilent Technologies Inc., Santa Clara, CA, USA). The cells were incubated overnight with primary antibodies in PBS at 4 °C. The cells were washed extensively in PBS and incubated at room temperature for 30 min with a secondary antibody. The nuclei were counterstained with 4′,6-diamidino-2-phenylindole (DAPI; diluted 1:500, #5748, FUJIFILM Wako Pure Chemical) in PBS at room temperature for 30 min. To prevent fading during microscopy, the cells were mounted in DakoCytomation fluorescent mounting medium (#S302380-2, Agilent Technologies Inc.). Immunofluorescence images were visualized and recorded using a Biorevo BZ-9000 fluorescence microscope (Keyence Corporation, Osaka, Japan). Quantifications of PBs carried out in triplicated experiments by blinded independent researchers, who chose five to seven fields in each session.

### RNA-seq and data analysis

Total RNA was extracted from cells using a Direct-zol RNA MiniPrep Kit (Zymo Research) as described above. The quantity and quality of RNA in the samples were confirmed by an Agilent 2100 Bioanalyzer using RNA Nano Chips (Agilent Technologies). Libraries for next-generation sequencing (NGS) were constructed using a TruSeq Stranded Total RNA Sample Prep LS Kit (#20020596, Illumina, San Diego, CA, USA) according to the manufacturer’s instructions. The constructed libraries were qualified and quantified by an Agilent 2100 Bioanalyzer using a high-sensitivity DNA assay (Agilent Technologies) and by quantitative PCR using a KAPA library quantification kit (#KK4824, Kapa Biosystems, Wilmington, MA). Finally, sequencing was performed at the NGS Core Facility at Kyoto Prefectural University of Medicine with HiScanSQ (Illumina, San Diego, CA, USA) using the standard 100 bp paired-end method. The produced RNA-seq base calls were converted into FASTQ file format by bcl2fastq2 conversion software v2.20 (Illumina). RNA-seq reads from the FASTQ files were then aligned to the reference mouse genome GRCm38/mm10 using TopHat2 v2.1.0 after performing quality control with FASTX Toolkit v0.0.13, FastQC v0.11.2, and PRINSEQ lite v0.20.4. Gene expression analysis was performed based on the fragments per kilobase million values calculated using the RNA-seq data by Cufflinks v2.2.1. The NGS data have been submitted to the Gene Expression Omnibus online database (http://www.ncbi.nlm.nih.gov/geo/) under accession number GSE166450.

### Statistical analysis

In gene expression analyses and quantification of oil red O staining, we performed triplicate experiments. All calculations were performed using Excel (Microsoft Office 2018, Redmond, WA, USA), and plots were created using Prism 8 (GraphPad Software Inc., San Diego, CA, USA). The results are expressed as the means ± SDs. The statistical significance of differences between the groups was evaluated using Student’s *t* test when the sample groups have equal variances or using Welch’s *t*-test when the groups have unequal variances, and *P* values < 0.05 were considered to indicate significance.

## Supplementary information

Supplementary Figure S1

Supplementary Figure S2

Supplementary Figure S3

Supplementary Figure S4

Supplemental Figure Legends
