# Supplementary figures and images for: RNA decay in processing bodies is indispensable for adipogenesis
Source: Cell Death Dis. 2021 Mar 17;12(4):285. doi: 10.1038/s41419-021-03537-7 (PMC7969960; doi:10.1038/s41419-021-03537-7)

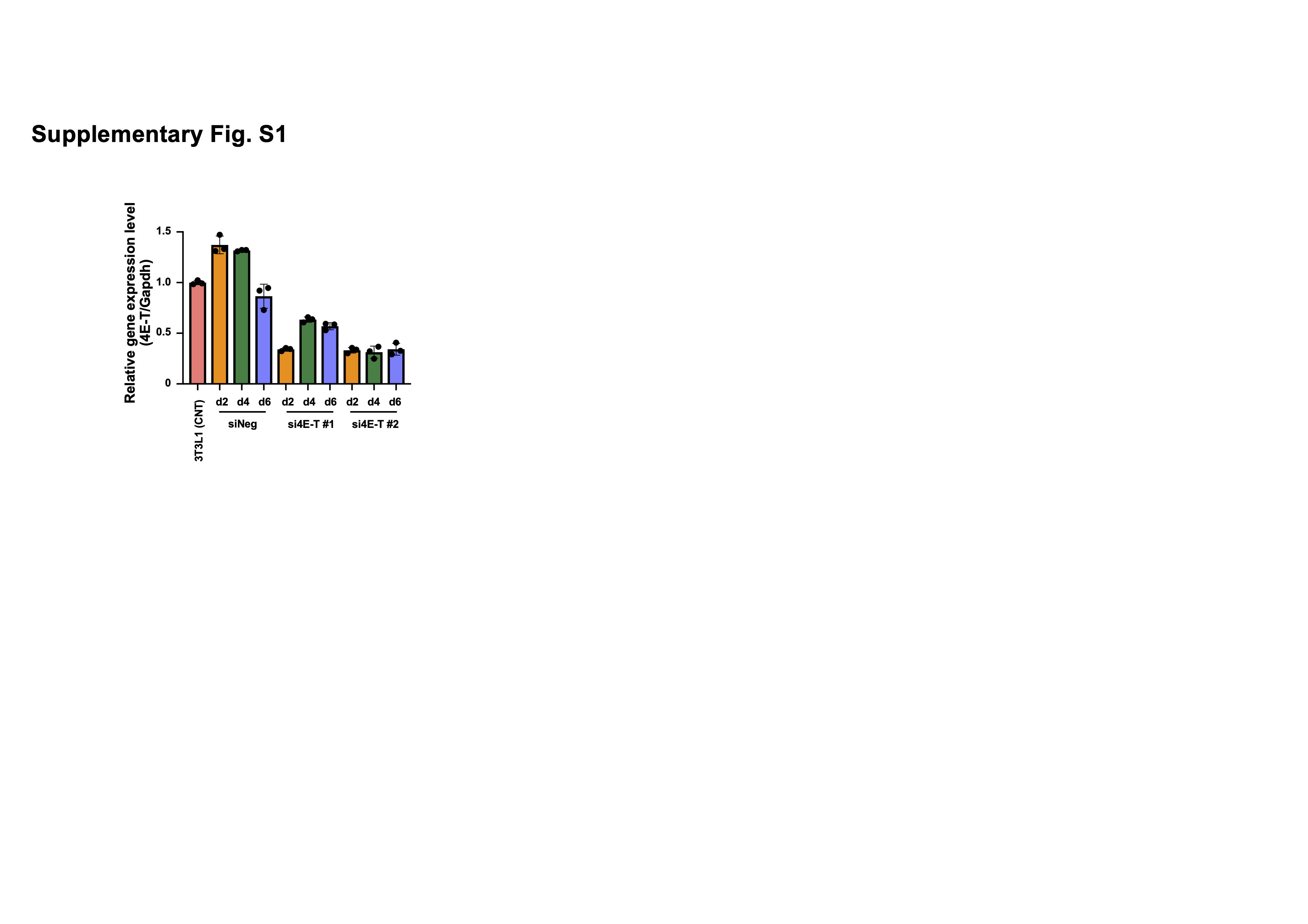

Supplement: Supplementary file 1 — Supplementary Figure S1 [file 41419_2021_3537_MOESM1_ESM.png]

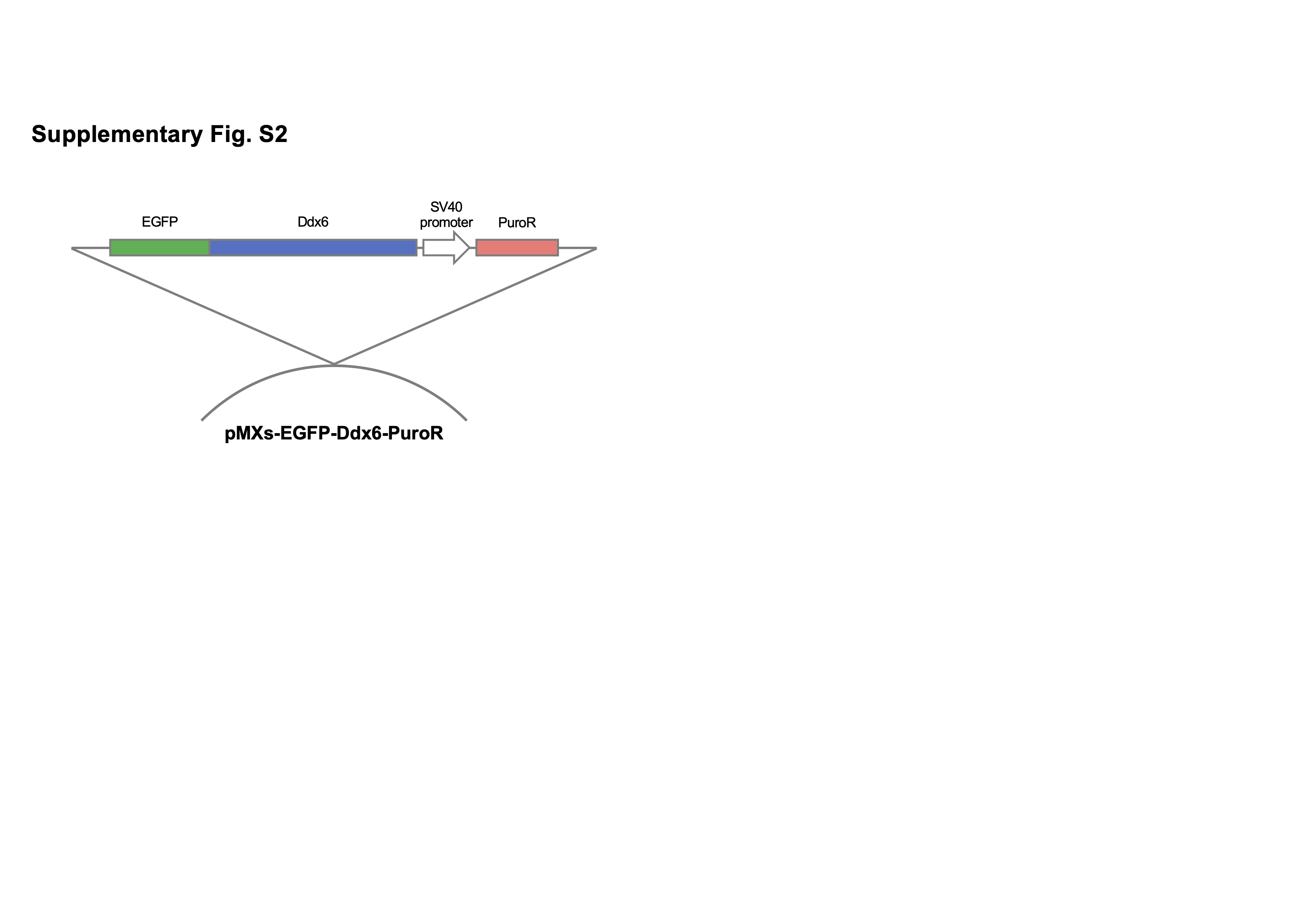

Supplement: Supplementary file 2 — Supplementary Figure S2 [file 41419_2021_3537_MOESM2_ESM.png]

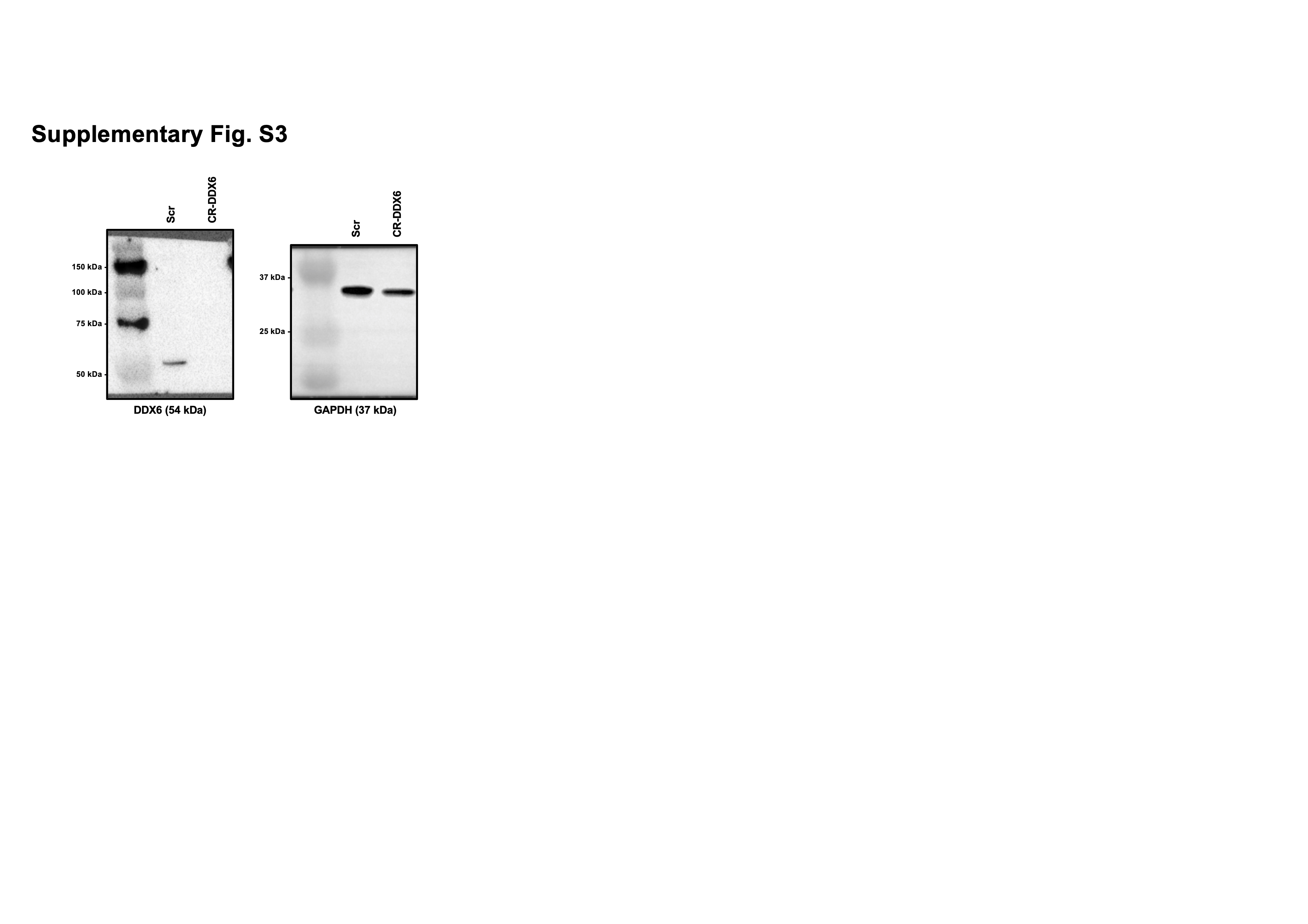

Supplement: Supplementary file 3 — Supplementary Figure S3 [file 41419_2021_3537_MOESM3_ESM.png]

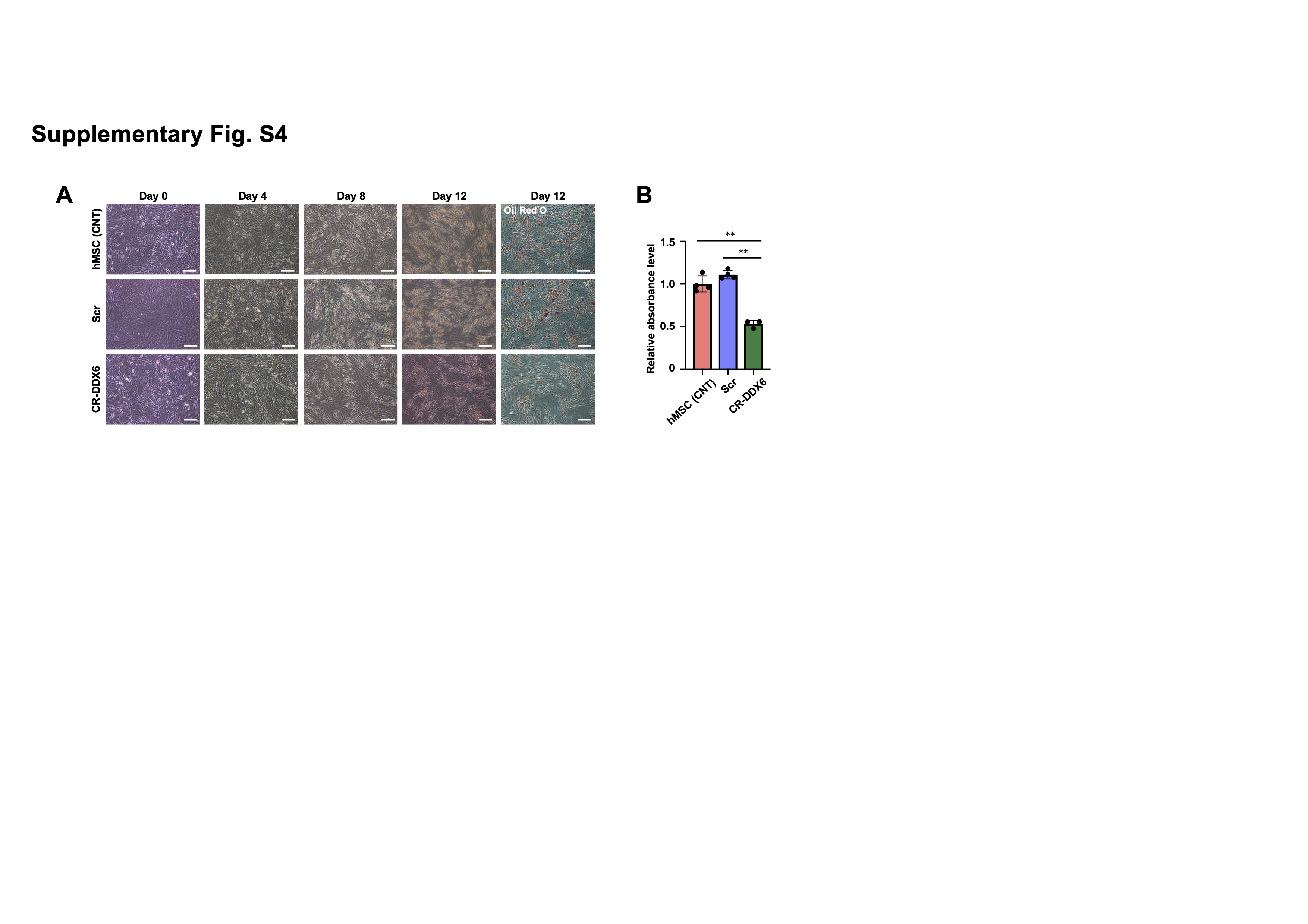

Supplement: Supplementary file 4 — Supplementary Figure S4 [file 41419_2021_3537_MOESM4_ESM.png]
