# Supplementary material for: RNA decay in processing bodies is indispensable for adipogenesis
Source: Cell Death Dis. 2021 Mar 17;12(4):285. doi: 10.1038/s41419-021-03537-7 (PMC7969960; doi:10.1038/s41419-021-03537-7)
Supplement: Supplementary file 5 — Supplemental Figure Legends [file 41419_2021_3537_MOESM5_ESM.docx]

Supplementary figure legends

Supplementary Fig. S1. RT-qPCR analysis of the expression of *4E-T* in 3T3L1 preadipocytes on each day.

Individual RNA expression levels were normalized to *Gapdh* expression levels. The error bars indicate the SDs (n = 3).

Supplementary Fig. S2. Overexpression of Ddx6.

For overexpression of Ddx6, Ddx6 was fused with EGFP. The sequence was inserted into the retroviral vector pMXs with a puromycin resistance gene.

Supplementary Fig. S3. DDX6 KO in hMSCs.

The DDX6 gene was knocked out using CRISPR/Cas9 technology in hMSCs. KO of DDX6 was verified by Western blotting. A scramble sequence was used as a negative control.

Supplementary Fig. S4. Adipogenic differentiation of DDX6-KO hMSCs.

(A) Phase-contrast microscopy images during adipogenesis. On day 12, cells were fixed and stained with Oil Red O. The scale bar is 100 μm. (B) Relative absorbance of Oil Red O. The error bars indicate the SDs (DDX6-KO: n = 3, control and scramble: n=4). ** indicates significance (P<0.01).
